# The sharing economy promotes sustainable societies

**DOI:** 10.1038/s41467-019-09260-4

**Published:** 2019-03-14

**Authors:** Zhifu Mi, D’Maris Coffman

**Affiliations:** The Bartlett School of Construction and Project Management, University College London, London, WC1E 7HB UK

## Abstract

A simultaneous improvement in both ecological and economic efficiency is necessary to achieve the Sustainable Development Goals (SDGs). The new sharing economy has potential to promote the needed shifts in collective consumption behaviour, but better governance models are urgently required.

## Abstract (web summary)

Sharing activities are under wide debate regarding the environmental impacts. Here the authors reviewed their benefits and problems and suggested that a simultaneous improvement of both ecological and economic efficiency is necessary to achieve the Sustainable Development Goals (SDGs).

The sharing economy is an emerging economic model usually defined as a peer-to-peer based sharing of access to goods and services, which are facilitated by a community-based online platform. It focuses on the sharing of underutilised assets in ways which improve efficiency, sustainability and community^1^. In economic theory, these platforms are essentially ‘club goods’ (a sub-type of public goods which are excludable but non-rivalrous), which have been characterised as an elegant Coasian solution, which reduces transaction costs to nearly zero whilst deterring free-riding^2,3^. The sharing model has been developing quickly in many service sectors, especially in transportation and hospitality.

While the sharing economy provides a potential pathway to sustainable societies, conflicts between business profits and social wellbeing can potentially arise. Service providers aim to maximize corporate profits to shareholders and value to paying customers, while governments aim to optimize wellbeing for all citizens^4^. There is a concern that some companies may use the ‘sharing economy’ as a marketing gimmick to disguise profit-motivation and exploitation under the pretence of making the society a better place. Governments on the other hand might be considered overly optimistic regarding the role such emerging business models can play in resolving a wealth of urban issues even in the absence of financial incentives or new regulations^4^. We suggest instead that such conflicts are resolvable through cooperation between sharing enterprises and governments. Public authorities should provide both economic and noneconomic incentives to private operators who have passed a complete Life Cycle Assessment (LCA) which estimates environmental impacts associated with all the stages of the shared product’s life (or over a firm or project’s lifecycle), while sharing service providers should take environmental protection and improvement of societal wellbeing as a Corporate Social Responsibility (CSR) rather than a marketing ploy.

The sharing economy has positive environmental impacts, through a reduction in the total resources required and it helps reduce pollutants, emissions and carbon footprints. In the transportation sector, vehicle sharing behaviour can have a positive environmental impact by decreasing the number of kilometers travelled. Such sharing activities can also stimulate long-lived changes in consumer behaviour by shifting personal transportation choices from ownership to demand-fulfilment. Similarly, bicycle sharing schemes can reduce the use of motorised vehicles that usually consume petroleum products and generate emissions. In Shanghai, bicycle sharing reduced carbon dioxide (CO_2_) and nitrogen oxide (NO_X_) emissions by 25,000 tonnes and 64 tonnes in 2016, respectively^5^.

The sharing of commodities and services can also provide health benefits^6^. The implementation of bike sharing programmes lead to greater propensities to cycle among individuals who live in regions where bike sharing services are available. Over ten thousand premature deaths are predicted to be avoided per year if all European cities reach a target of a quarter (25%) of all trips made by cycling^7^.

However, despite these clear benefits, some studies have cast doubts on the sharing economy’s environmental effectiveness and intrinsic sustainability^8,9^. Fishman et al.^10^ suggested that bike sharing can actually increase the overall motor vehicle usage if inventory management is not optimised or when the effects of bike re-distribution and maintenance are taken into consideration. The phenomenon can be readily observed from the reckless use of dockless bike-sharing in China^11^. In order to snatch market share, sharing companies have placed excessive dockless cycles in many Chinese cities.

In light of such ‘side-effects’ the sharing economy will only prove sustainable through the mutual cooperation amongst public authorities, enterprises and consumers (Fig. 1)^12^. Firstly, governments have a role to play in identifying the sharing models that are most pro-social from a LCA perspective and supporting service providers through both economic (e.g. lower taxes and subsidies) and noneconomic incentives (e.g. communication campaigns and labelling). In following such an approach, both environmental and health benefits can be converted into economic incentives for enterprises. In addition, governments need to monitor emerging sharing models to mitigate against excessive provision of sharing services, which has happened in many fields as companies competed for market share.

**Fig. 1:**
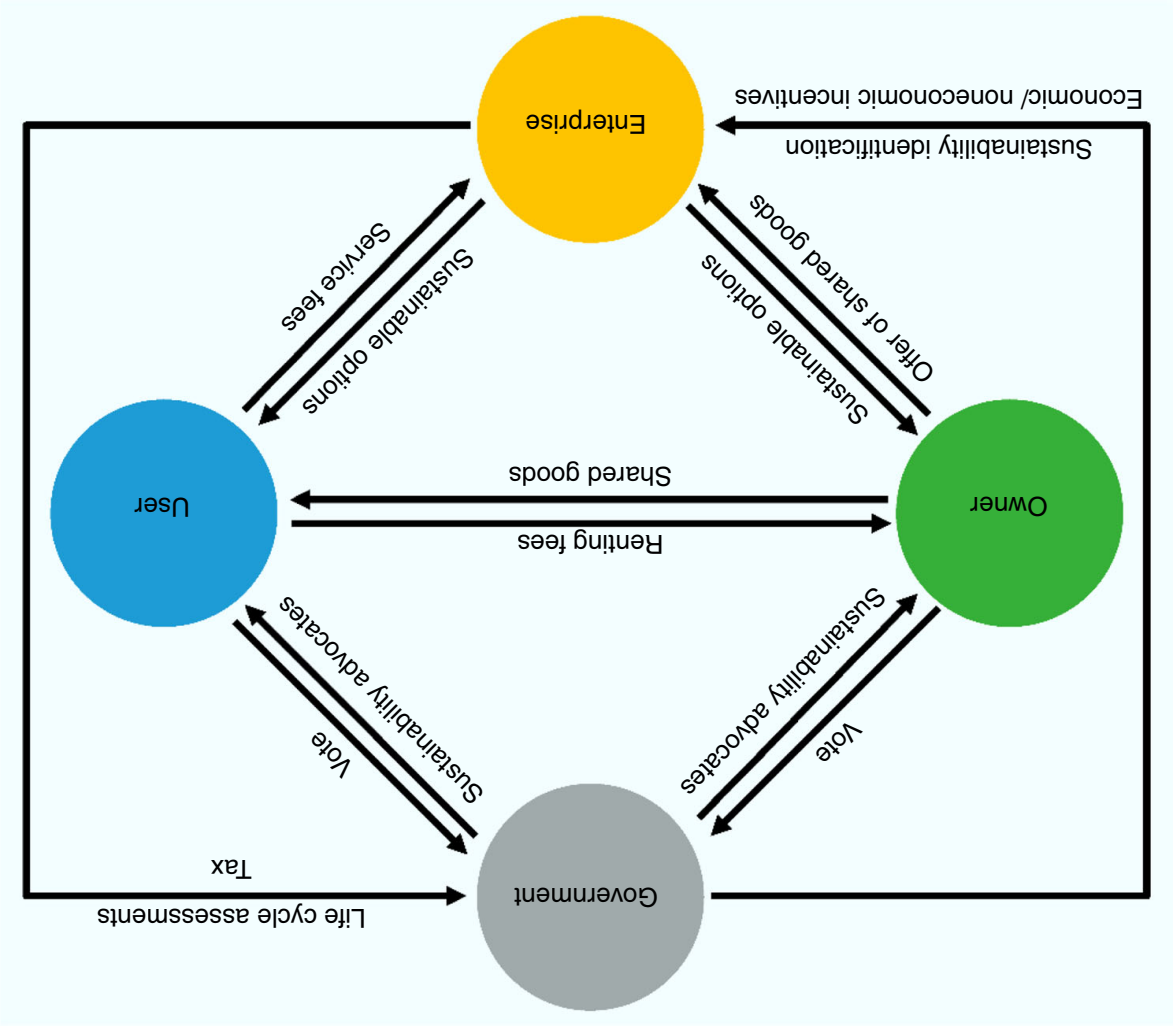
Framework for sustainable model of sharing economy. The framework involves four participants, including governments, enterprises, owners and users. Governments identify the most pro-social sharing models and support service providers through both economic and noneconomic incentives. Enterprises estimate the environmental impacts of sharing activities from life-cycle perspectives and provide sustainable information and options to consumers. Owners and users of shared products make sustainability a key factor in selecting sharing service providers

Secondly, sharing enterprises must set the sustainability of sharing models as an objective rather than its use as a marketing tool. They should commit to continuous development of shared goods so that they can meet the standards set by the government regarding LCA and can obtain or maintain eligibility for incentives. The aim of LCA is to compare the full range of environmental impacts of the shared products and services, and this information is useful to improve efficiency and support policy. The tax system on sharing activities should to be improved, as tax evasion is very common in sharing economy. Governments should make it a priority to use the tax collected from sharing activities to promote sustainability, by providing incentives to sharing enterprises and sustainability advocates to consumers.

Thirdly, as well as governments and enterprises, consumers—owners and users of shared products—play a critical role in the sustainability of sharing economy, as they are the most essential participants in the sharing economy. Enterprises need to provide sustainable information and options to consumers, and governments need to encourage consumers to select more sustainable goods and services. Successful strategies for incentivising enterprises and nudging consumers will depend on cultural contexts, but can include tax breaks to firms to encourage compliance and partnerships with health, life and auto insurance companies to encourage participation in appropriate bike or ride sharing schemes by offering premium reductions for behaviour that improves health, for example, or reduces congestion (and thus risk of collision). Consumers need to enhance their environmental awareness and make sustainability a key factor in selecting sharing service providers and in voting for politicians who are prepared to demand corporate social responsibility from the sharing economy.

Sharing enterprises also need to develop relationships with the local authorities and follow the related regulations in order to achieve long-term viability. Here what is needed is more explicit acknowledgement by local and national governments of the importance of the sharing economy for achieving SDGs; the challenge is to better align the interests of both new and old businesses, local governments, and the national economy. Unfortunately, what too often happens is that these new sharing platforms, with their rhetoric of disruption, position themselves as orthogonal both to the state and the market (old businesses) in sweeping away inefficient business models and upending old regulatory regimes. Policymakers have to respond by partnering with pro-social solutions, offering them local government support in exchange for their assistance in mitigating the costs associated with structural change.

Over the long-term, to ensure the viability and resilience of sharing firms, their capital structures should be considered carefully. Early community ride-sharing services, when they proved successful, often had to be sold to car rental companies to maintain growth at scale. Most of the current players are privately held, and will soon be taken public, possibly with outsized valuations. If the ‘Unicorn Bubble’ bursts and these initial public offerings (IPOs) fail, a shakeout is inevitable, with some firms emerging as Amazons and Ebays. In the very short-term, state actors may be well-advised to wait until clear winners emerge, as excessive leverage can end up harming stakeholders as well as shareholders^13^. Alternatively, state actors might use concessions and public procurement systems as a means of identifying those firms that are best aligned with specific sustainability goals or who score highest in the LCA measures.

At the same time, while sharing service providers play a crucial economic role in coordinating and reducing transaction costs to both customers and suppliers who are independent economic agents voluntarily participating in a transaction, current platforms tend to tilt consumer communication towards the interests of customers without adequate regard for the interests of equally vulnerable service suppliers. The role of supply chains in sharing firms is largely under-researched, primarily because the sharing economy presupposes a peer-to-peer model that does not always exist in practice. Most bike sharing firms own the fleets of bicycles (rather than allowing individuals to share their own bicycles during idle hours), and as firms scale-up operations, their business models change, e.g. platforms, like Ebay, originally designed to facilitate sale of used goods, now primarily markets new merchandise, some of which was even manufactured expressly to be sold on Ebay. In order to assess the carbon footprints of sharing economy firms accurately, their supply chains must be examined in detail.

From the perspective of achieving the UN’s SDGs, the sharing economy’s benefits of increased economic efficiency and reduced information asymmetries can scarcely be considered untoward developments, but the need for better coordination of national and local government and a direct engagement with the firms themselves is key to addressing dislocations and negative externalities. Regulatory parity and public safety must command the attentions of state actors. Over time, the sharing economy will also help foster structural change towards lower carbon economies, but the empirical evidence for these effects is, as yet, mixed, and there is a great deal of room for optimisation strategies, just as there is a pressing need for more research to inform evidence-based policy-making.
